# A Magnesium‐Enriched 3D Culture System that Mimics the Bone Development Microenvironment for Vascularized Bone Regeneration

**DOI:** 10.1002/advs.201900209

**Published:** 2019-04-18

**Authors:** Sihan Lin, Guangzheng Yang, Fei Jiang, Mingliang Zhou, Shi Yin, Yanmei Tang, Tingting Tang, Zhiyuan Zhang, Wenjie Zhang, Xinquan Jiang

**Affiliations:** ^1^ Department of Prosthodontics Shanghai Engineering Research Center of Advanced Dental Technology and Materials Shanghai Research Institute of Stomatology National Clinical Research Center for Oral Diseases Shanghai Key Laboratory of Stomatology Ninth People's Hospital College of Stomatology Shanghai JiaoTong University School of Medicine 639 Zhizaoju Road Shanghai 200011 P. R. China; ^2^ Department of Orthopaedic Surgery Ninth People's Hospital Shanghai JiaoTong University School of Medicine 639 Zhizaoju Road Shanghai 200011 P. R. China; ^3^ Department of Oral and Maxillofacial‐Head and Neck Oncology Shanghai Research Institute of Stomatology National Clinical Research Center for Oral Diseases Shanghai Key Laboratory of Stomatology Ninth People's Hospital College of Stomatology Shanghai JiaoTong University School of Medicine 639 Zhizaoju Road Shanghai 200011 P. R. China

**Keywords:** 3D culture systems, biomaterials, developmental microenvironment, magnesium, vascularized bone regeneration

## Abstract

The redevelopment/regeneration pattern of amputated limbs from a blastema in salamander suggests that enhanced regeneration might be achieved by mimicking the developmental microenvironment. Inspired by the discovery that the expression of magnesium transporter‐1 (MagT1), a selective magnesium (Mg) transporter, is significantly upregulated in the endochondral ossification region of mouse embryos, a Mg‐enriched 3D culture system is proposed to provide an embryonic‐like environment for stem cells. First, the optimum concentration of Mg ions (Mg^2+^) for creating the osteogenic microenvironment is screened by evaluating MagT1 expression levels, which correspond to the osteogenic differentiation capacity of stem cells. The results reveal that Mg^2+^ selectively activates the mitogen‐activated protein kinase/extracellular regulated kinase (MAPK/ERK) pathway to stimulate osteogenic differentiation, and Mg^2+^ influx via MagT1 is profoundly involved in this process. Then, Mg‐enriched microspheres are fabricated at the appropriate size to ensure the viability of the encapsulated cells. A series of experiments show that the Mg‐enriched microenvironment not only stimulates the osteogenic differentiation of stem cells but also promotes neovascularization. Obvious vascularized bone regeneration is achieved in vivo using these Mg‐enriched cell delivery vehicles. The findings suggest that biomaterials mimicking the developmental microenvironment might be promising tools to enhance tissue regeneration.

## 1 Introduction

Tissue defects caused by accidents and diseases severely decrease quality of life, and humans possess limited regenerative capacity; thus, the repair of large bone defects remains a major clinical challenge.^1^ Some animals, such as the salamander, can regrow a whole limb after amputation.^2^ Study of the mechanism revealed that the amputated limb regenerates from the blastema and that the involved cellular and molecular mechanisms are highly similar to embryonic development processes.^3^ Advances in stem cells and biomaterials have improved artificially assisted tissue regeneration strategies that provide new hope for patients.^4^ Based on the redevelopment/regeneration model in salamander, we believe that the construction of an artificial embryonic‐like microenvironment for stem cells would be a promising strategy for regenerative medicine that may achieve better regenerative effects.

**Figure 1:**
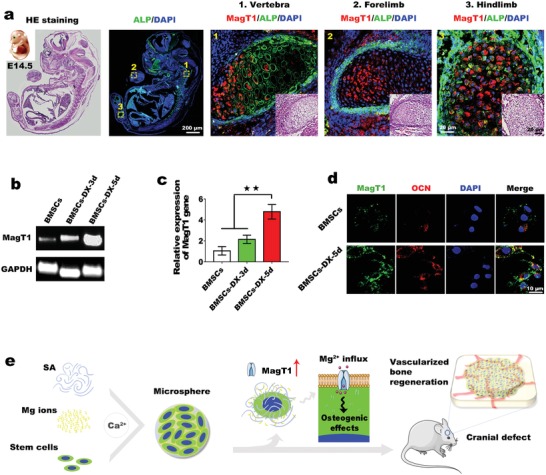
a) Colocalization of ALP and MagT1 in mouse embryos using double immunofluorescence staining. b) DNA gel electrophoresis analysis of MagT1 expression in BMSCs incubated with or without osteogenic medium (DX). c) qPCR shows the upregulation of MagT1 during osteogenic induction (*n* = 3, ** *p* < 0.01). d) Double immunofluorescence staining of OCN and MagT1. e) Schemata of construction of the Mg‐enriched 3D culture system for bone regeneration. Mg^2+^ influx via MagT1 mediates the osteogenic differentiation of stem cells.

Biomaterial strategies for bone regeneration have gained remarkable attention in terms of the repair of large bone defects.^5^ Previous studies have reported that modification of the structure and composition of biomaterials to mimic natural bone tissue has improved bone regeneration with considerable efficacy.^6^, ^7^ For instance, the incorporation of bioactive ions present in natural bone matrix, such as copper ions, strontium ions, and magnesium ions (Mg^2+^), into bone substitutes stimulates vascularized bone regeneration.^8^ However, the specific mechanisms underlying the stimulatory effects of these bioactive ions are not fully understood. Through studies involving mouse embryos, we discovered a novel phenomenon in which magnesium transporter‐1 (MagT1), one of the major Mg^2+^ transporters, was selectively and highly expressed in the endochondral ossification region (**Figure** **1**a). We also detected similar high MagT1 expression in rat bone marrow stem cells (BMSCs) undergoing osteogenic differentiation (Figure 1b–d). It is well documented that endochondral ossification is an innate vascularized bone formation process, suggesting that MagT1 expression might be exploited for vascularized bone regeneration.^9^, ^10^ It is worth noting that Mg^2+^ released from biomaterials has been reported to induce the upregulation of MagT1 in rat BMSCs.^11^ Therefore, we hypothesize that local Mg^2+^ accumulation leading to high MagT1 expression might mimic the bone development microenvironment and thus induce vascularized bone formation. However, the optimum concentration of Mg^2+^ for constructing the angiogenic and osteogenic microenvironments is unclear, and the signaling pathways downstream of Mg^2+^ entry via MagT1 have not yet been elucidated. These issues highlight the acute demand for further investigation to elucidate the specific effects of MagT1 on vascularized bone regeneration.

In the present study, inspired by the discovery that MagT1 expression was significantly upregulated in the endochondral ossification region, we identified the optimum concentration of Mg^2+^ for constructing the osteogenic microenvironment by altering the expression level of MagT1, which is consistent with the osteogenic differentiation capacity of BMSCs. A series of experiments proved that Mg^2+^, through MagT1, not only promotes the osteogenic differentiation of BMSCs through activation of the mitogen‐activated protein kinase/extracellular regulated kinase (MAPK/ERK) pathway but also enhances neovascularization by promoting the migration of endothelial cells (ECs). Based on these findings, we developed a Mg‐enriched 3D culture system to mimic the bone development microenvironment for vascularized bone regeneration (Figure 1e). Marked vascularized bone regeneration was achieved after implantation of the cell delivery vehicles into rat critical‐sized cranial defects. Taken together, the data suggest that our bioinspired regeneration method of constructing an embryonic‐like microenvironment for stem cells is a promising strategy for bone regeneration.

## 2 Results and Discussion

### 2.1 MagT1 Expression Is Strongly Correlated with Embryonic Bone Development and Osteogenic Differentiation

To explore the correlation of MagT1 expression with embryonic bone development, colocalization analysis of alkaline phosphatase (ALP), an osteogenic marker, and MagT1 was carried out in mouse embryos at embryonic day (E) 14.5 using a double immunofluorescence staining technique. As shown in Figure 1a, there was selective high MagT1 expression in the ALP‐positive region in the cartilage anlages of both limbs and vertebrae. MagT1 is a major Mg transporter that influences intracellular Mg homeostasis. Previous studies elucidated that MagT1‐dependent Mg^2+^ entry significantly affects various cell behaviors, including cell proliferation, differentiation and T‐cell‐specific immunomodulation,^12^, ^13^ but there are no relevant reports on its correlation with embryonic bone development. Herein, we found that MagT1 is highly expressed during endochondral bone formation. Based on this finding, we further investigated MagT1 expression in rat BMSCs during osteogenic differentiation induced by osteogenic medium containing dexamethasone (DX). Changes in MagT1 gene expression were assessed by DNA gel electrophoresis and quantitative polymerase chain reaction (PCR) (qPCR) over the following 5 day period. MagT1 expression levels slightly increased in BMSCs induced for 3 days and significantly increased in BMSCs induced for 5 days compared to untreated BMSCs (Figure 1b,c). In addition, we detected MagT1 protein levels by immunofluorescence staining. Osteocalcin (OCN) was used to measure the effectiveness of osteogenic induction. OCN expression was relatively low in undifferentiated BMSCs but increased markedly after 5 days of induction. At the same time, MagT1 expression in differentiated cells showed a consistent upregulation (Figure 1d). Based on these findings, we believe that MagT1 is profoundly involved in osteogenesis. Moreover, endochondral ossification is a vascularized bone formation process, indicating that MagT1 expression might be important for vascularized bone regeneration. Notably, a recent study reported that an increase in extracellular Mg could upregulate MagT1 expression in BMSCs.^11^ In addition, the local accumulation of Mg^2+^ has positive effects on osseointegration and osteogenesis.^14^ We thereby hypothesized that a Mg‐enriched environment might simulate the bone development microenvironment to promote vascularized bone formation via upregulating MagT1.

### 2.2 The Mg‐Enriched Environment Induces BMSC Osteogenic Differentiation via High MagT1 Expression

**Figure 2:**
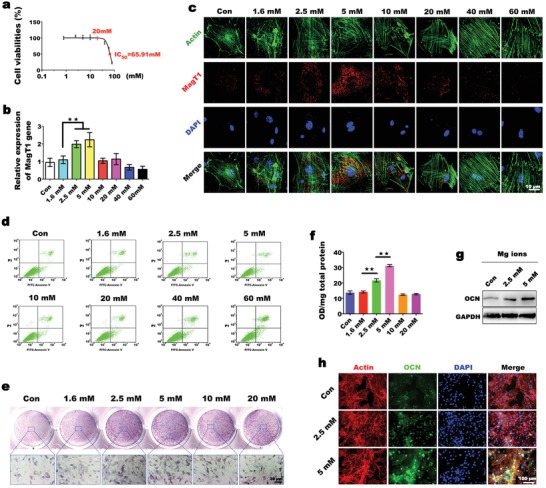
Mg^2+^ promotes osteogenic differentiation via high MagT1 expression. a) Analysis of the effect of Mg^2+^ on cell viability using IC_50_ assays (*n* = 6). b) qPCR analysis of MagT1 expression in BMSCs treated with different concentrations of Mg^2+^ (*n* = 3, **p < 0.01). c) Immunofluorescence assay of MagT1 expression in BMSCs after 5 days of incubation. The cytoskeletal structure (green) and nuclei (blue) were stained separately. d) Flow cytometry‐based apoptosis assay using Annexin V‐FITC/PI staining. Dots in the upper right quadrant represent late apoptotic cells. e) ALP staining of BMSCs exposed to different Mg^2+^ environments for 5 days. f) Semiquantitative analysis of ALP activity after 5 days of induction (*n* = 3, **p < 0.01). g) Immunoblots display the increased expression of OCN in BMSCs induced by a Mg‐enriched environment. h) Detection of OCN levels by immunofluorescence staining.

To investigate whether the Mg‐enriched environment stimulates osteogenic differentiation by mimicking the bone development microenvironment with high MagT1 expression, BMSCs were cultured in Mg‐containing medium. First, we cultured BMSCs with medium containing varying concentrations of Mg^2+^ to determine the appropriate Mg environment for cell survival. According to the half maximal inhibitory concentration (IC_50_) assays, cell viability was unaffected at Mg^2+^ concentrations of less than 20 × 10^−3^ m but decreased significantly when the concentration exceeded 20 × 10^−3^ m. When the concentration reached 65.91 × 10^−3^ m, half of the cells were apoptotic (**Figure** **2**a). A consistent trend was observed when detecting apoptosis by flow cytometry. Compared with cells cultured in normal medium (0.8 × 10^−3^ m Mg^2+^), BMSCs exposed to Mg^2+^ concentrations above 20 × 10^−3^ m showed a significant increase in the late apoptotic or necrotic population (Annexin V‐FITC+ and propidine iodide (PI)+) (Figure 2d). Based on these results, Mg^2+^ concentrations under 60 × 10^−3^ m were selected for subsequent experiments. The effects of Mg^2+^ on osteogenic differentiation vary with concentration. Low extracellular Mg^2+^ concentration (i.e., 0.1 × 10^−3^ m) inhibits the osteogenic differentiation of BMSCs, while a high Mg^2+^ concentration (i.e., 18 × 10^−3^ m) has detrimental effects on osseous metabolism.^15^ Hence, the appropriate Mg environment is pivotal in terms of bone formation. However, the optimum concentration for the induction of MagT1 expression and osteogenic differentiation was unclear. Therefore, we used MagT1 expression in BMSCs as a criterion for optimizing the Mg^2+^ concentration for osteogenic induction. As the Mg^2+^ concentration increased, MagT1 expression generally showed an increasing trend and then gradually decreased to the control level when the Mg^2+^ concentration exceeded 10 × 10^−3^ m (Figure 2b,c). Notably, MagT1 expression was markedly increased in the 2.5 × 10^−3^ and 5 × 10^−3^ m groups, especially in the 5 × 10^−3^ m group. As mentioned above, the upregulation of MagT1 is closely related to the osteogenic differentiation of BMSCs. We therefore hypothesized that Mg^2+^ exerts osteogenic inductive effects below 10 × 10^−3^ m, and concentrations from 2.5 × 10^−3^ to 5 × 10^−3^ m are optimal for osteogenic induction. To verify this hypothesis, BMSCs were incubated in medium containing Mg^2+^ at concentrations less than 20 × 10^−3^ m. Osteogenic induction by Mg^2+^ was evaluated based on ALP activity. The trend in ALP activity induced by Mg^2+^ was consistent with that in MagT1 expression, suggesting that MagT1 expression is consistent with the osteogenic differentiation capacity of BMSCs (Figure 2e,f). To further evaluate the osteogenic inductive effects of 2.5 × 10^−3^ and 5 × 10^−3^ m Mg^2+^, the protein levels of OCN were detected after cell treatment. Compared with the control group, the Mg‐enriched groups showed an obvious increase in OCN protein levels, particularly in the 5 × 10^−3^ m group (Figure 2g,h). These findings confirmed that a Mg‐enriched environment could stimulate osteogenic differentiation by mimicking the bone development microenvironment with high MagT1 expression, and a concentration of Mg^2+^ ranging from 2.5 × 10^−3^ to 5 × 10^−3^ m is optimal for constructing an osteogenic microenvironment.

### 2.3 Molecular Mechanisms Underlying the Osteogenic Inductive Effects of Mg^2+^

**Figure 3:**
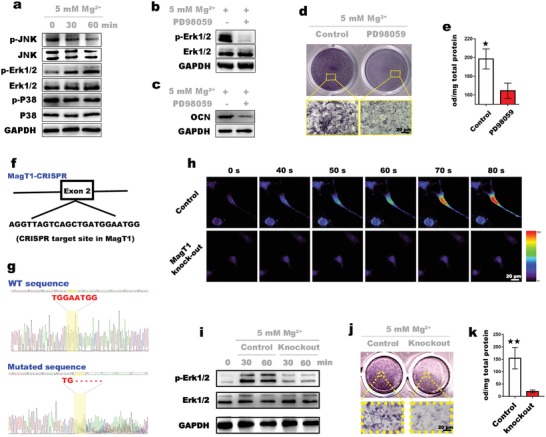
Investigation of the mechanisms underlying the osteoinductivity of Mg^2+^. a) Western blot assay shows the phosphorylation of MAPK pathway components in BMSCs treated with 5 × 10^−3^ m Mg^2+^. b) Immunoblots display the inhibitory effects of PD98059, a specific Erk1/2 inhibitor, on the activation of Erk1/2 (60 min) induced by Mg^2+^. c) An obvious decrease in OCN levels in BMSCs treated with PD98059 was detected by western blotting. d) ALP staining of BMSCs treated with or without PD98059. e) Semiquantitative analysis of ALP activity in BMSCs (*n* = 3, *p < 0.05). f) Site targeted by the CRISPR/Cas9 system in the rat MagT1 gene. g) A 6 bp mutation (in yellow area) at the target site resulting from CRISPR/Cas9 targeting was confirmed by gene sequencing. h) Mg–Fura‐2 detection of Mg^2+^ entry into BMSCs. i) Inhibitory effects of MagT1 knockout on Erk1/2 activation. j) ALP staining of normal and mutated BMSCs incubated with 5 × 10^−3^ m Mg^2+^ for 5 days. k) Semiquantitative analysis shows a sharp decrease in ALP activity in mutated BMSCs (*n* = 3, **p < 0.01).

The mechanisms by which Mg^2+^ promotes osteogenic differentiation have not been completely clarified. In the present study, we focused on the effects of Mg^2+^ on the MAPK pathway, one of several signaling pathways governing the osteogenic differentiation of stem cells.^16^ First, BMSCs were cultured in medium containing 5 × 10^−3^ m Mg^2+^. Samples were collected at different time points and prepared for western blot analysis. As shown in **Figure** **3**a, Mg^2+^ significantly enhanced the phosphorylation of Erk1/2 at 30 and 60 min. In contrast, there were no significant changes in the phosphorylation of other members of the MAPK pathway, i.e., P38 and c‐Jun N‐terminal kinase (JNK), implying that Mg^2+^ stimulates osteogenic differentiation through selective activation of the MAPK/ERK pathway. The Erk1/2‐specific inhibitor PD98059 was applied to verify whether Mg^2+^ promotes osteogenic differentiation via the Erk1/2 signaling pathway. As shown in Figure 3, Mg^2+^‐induced OCN expression and APL activity were significantly depressed (Figure 3c–e) upon inhibition of Erk1/2 phosphorylation (Figure 3b). These results supported our hypothesis that Mg^2+^ promotes osteogenic differentiation through selective activation of the Erk1/2 signaling pathway. It is worth noting that abrogation of Mg^2+^ influx due to MagT1 deficiency impairs cellular activation signals.^12^ Therefore, we speculated that MagT1 affects osteogenic differentiation by regulating Mg^2+^ influx. Briefly, CRISPR/Cas9‐mediated targeted disruption of MagT1 was conducted to explore the regulatory role of MagT1 in Mg‐induced osteogenic differentiation. The Cas9 sgRNA‐targeted site is shown in Figure 3f. Sequencing analysis identified the mutation in the MagT1 gene (Figure 3g; Files S1 and S2, Supporting Information). To ascertain whether MagT1 knockout affects Mg uptake, we used a Mg^2+^‐sensitive dye, Mg‐Fura‐2, to monitor intracellular Mg^2+^ levels in BMSCs. The addition of Mg^2+^ induced a notable increase in intracellular fluorescence intensity in the control group, indicating Mg^2+^ entry into BMSCs, while only a slight change was observed in MagT1‐knockout BMSCs (Figure 3h; Movies S1 and S2, Supporting Information). The results suggested that cellular Mg^2+^ uptake decreases markedly upon MagT1 gene disruption. Moreover, by western blot analysis, a significant decrease in Erk1/2 phosphorylation consequent to MagT1 gene knockout was evident (Figure 3i). Correspondingly, MagT1 gene knockout significantly downregulated ALP activity in BMSCs after 5 days of induction with Mg^2+^ (Figure 3j,k), indicating a considerable depression of osteogenic differentiation. These results again highlighted that MagT1 expression is consistent with the osteogenic differentiation capacity of BMSCs. Taken together, the results indicated that Mg^2+^ induces the osteogenic differentiation of stem cells through selective activation of the MAPK/ERK pathway, and the osteoinductive effect is closely related to Mg^2+^ influx mediated by MagT1.

### 2.4 Fabrication and Optimization of a Mg‐Enriched 3D Culture System

**Figure 4:**
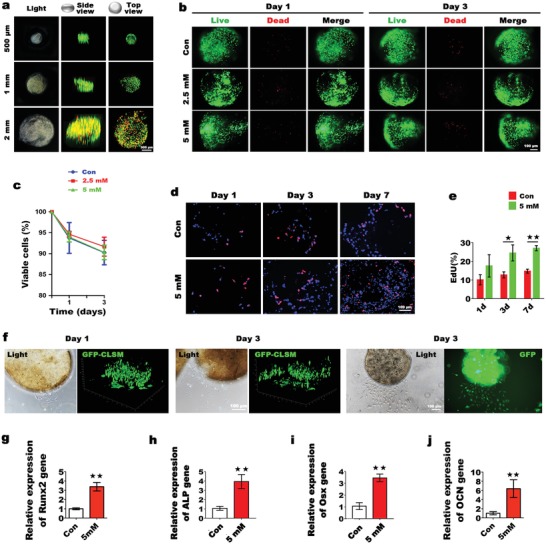
Optimization of the Mg‐enriched 3D culture system and the in vitro osteogenic differentiation of encapsulated BMSCs. a) Cell viability analysis of BMSCs inside spheroids of different sizes. Live cells (green) and dead cells (red) were detected by CLSM. b) BMSCs remained viable inside spheroids with a localized Mg‐enriched microenvironment. c) CCK‐8 assays of the viability of encapsulated BMSCs at different time points (*n* = 3, 50 µL of spheroids per sample). d) Sequential evaluation of encapsulated BMSC proliferation by EdU staining. e) Percentage of EdU‐positive cells encapsulated in spheroids (*n* = 3, *p < 0.05, **p < 0.01). f) Observation of GFP‐labeled cell migration from spheroids to culture dishes. 3D images were reconstructed by CLSM. g–j) The graphs show that the local Mg‐enriched environment in spheroids significantly stimulated the expression of Runx2 and ALP in BMSCs at the early stage of incubation (day 3), while high Osx and OCN expressions were observed at day 5 (*n* = 3, 50 µL of spheroids per sample. **p < 0.01).

After optimizing the concentration of Mg^2+^ for osteogenic induction, we constructed a Mg‐enriched 3D culture system to mimic the bone development microenvironment. Mg^2+^ can form physical crosslinks with sodium alginate (SA) molecules.^17^ Therefore, we created a locally Mg‐enriched environment using SA hydrogel to immobilize Mg^2+^. During embryonic development, cells tend to aggregate into spheres to form complex tissues with 3D structures and strong cell–cell interactions.^18^ In vitro, this process of self‐assembly also occurs when cells cannot attach to the surface of materials to reinforce intercellular connections.^19^ In short, the spheroid cell culture system simulates the tissue development environment. We thereby designed a spheroid 3D culture system. Briefly, BMSCs were suspended in medium containing a high Mg^2+^ concentration, and the cell suspension was mixed with SA solution. Then, the mixture was dropped into CaCl_2_ solution to form spheroids. Notably, cells inside microspheres, especially those in deep area, are susceptible to inadequate nutrient and oxygen supply. Research indicates that a central necrosis arises when the diameter of cellular tumor spheroid reaches over 600 µm.^20^ Therefore, selecting an appropriate microsphere size is important for cell survival. To determine the optimal size of spheroids for cell survival, spheroids of three different sizes were fabricated: small (spheroid diameter: 500 µm), medium (spheroid diameter: 1 mm), and large (spheroid diameter: 2 mm). Cell viability was measured by Live/Dead assays. As shown in **Figure** **4**a, cells were evenly distributed in spheroids. After 3 days of culture, only a few dead cells (stained in red) were found in the small group, while the number of dead cells in the microsphere core gradually increased with increasing size. When the diameter of the microspheres reached 2 mm, the cells in the microsphere core showed obvious deterioration. Therefore, we fixed the diameter of the microspheres at 500 µm. Noteworthy, for further fine‐tuning the size and morphology of the spheroids that ensure the cell viability, biofabrication techniques such as electrospraying could be employed.^21^

### 2.5 A Mg‐Enriched Environment Stimulates the Osteogenic Differentiation of Encapsulated BMSCs by Mimicking the Bone Development Microenvironment

Stem cell survival, proliferation, and migration are important for osteogenic differentiation.^22^ To evaluate the suitability of Mg‐enriched spheroids as an in vitro cell culture platform and in vivo biomimetic scaffolds for bone regeneration, we first assessed the viability of BMSCs inside the spheroids. Briefly, three groups of cell‐laden spheroids were fabricated: spheroids with local Mg^2+^ concentrations of 2.5 × 10^−3^ and 5 × 10^−3^ m (experimental groups) and spheroids with a normal Mg^2+^ concentration (0.8 × 10^−3^ m). As shown in Figure 4b, hardly any dead cells were found in the three groups after 1 day of culture, the number of dead cells increased slightly after 3 days of culture, and no significant difference was observed among the three groups. The CCK8 assay results showed that cell viability of >90% was maintained in each group for 3 days, demonstrating the innate biocompatibility of the Mg‐enriched microspheres (Figure 4c). Mg^2+^ concentrations ranging from 1.6 × 10^−3^ to 20 × 10^−3^ m were shown to enhance cell proliferation in vitro (Figure S1, Supporting Information); therefore, we examined whether the Mg‐enriched environment of spheroids stimulated cell proliferation as well. 5‐ethynyl‐2′‐deoxyuridine (EdU) staining was performed at different time points. At day 3, there were clear differences in the number of EdU‐positive cells (stained in pink) between the experimental group and the control group that became more significant at day 7, although all groups showed an increase in EdU‐positive cells over time. Overall, the Mg‐enriched environment stimulated the proliferation of encapsulated BMSCs (Figure 4d,e). Moreover, significant migration of the stem cells from the spheroids to the culture dishes was observed. Briefly, BMSCs were labeled with green fluorescent protein (GFP), and the spatial distribution of the cells was detected by a confocal laser scanning microscope (CLSM). As shown in Figure 4f, only a few cells migrated out of the microspheres after 1 day of culture. At day 3, sufficient cells had migrated out of the spheroids to enable the growth of large cell populations on the culture dishes, which is pivotal for BMSCs to participate in the regeneration process in vivo. To investigate the effect of the Mg‐enriched environment on the osteogenic differentiation of BMSCs, the expression levels of several osteogenesis‐related genes were detected by qPCR. The expression of Runt‐related transcription factor 2 (Runx2) and ALP increased significantly after 3 days of culture. After 5 days, the expression levels of Osterix (Osx) and OCN in BMSCs increased significantly by 3.5‐fold and by 6.4‐fold, respectively, in the experimental group compared with the control group. Overall, we believe that Mg‐enriched spheroids successfully simulate the bone development microenvironment, and the localized Mg‐enriched environment has considerable positive effects on the proliferation and migration of encapsulated BMSCs and stimulates osteogenic differentiation.

### 2.6 In Vitro and In Vivo Analyses of the Angiogenic Effects of the Mg‐Enriched Environment

**Figure 5:**
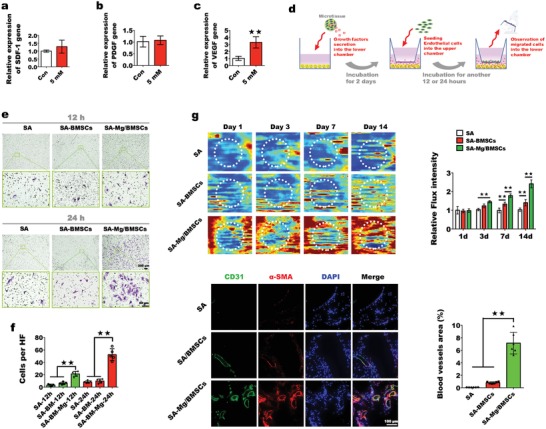
In vitro and in vivo analyses of the angiogenic effects of a Mg‐enriched microenvironment. a–c) qPCR analysis of the expression levels of chemotactic cytokine genes (*n* = 3, **p < 0.01). d) Schemata of the transwell migration model. e) Cells that migrated to the lower chamber were visualized by microscopy after crystal violet staining (*n* = 3, **p < 0.01). f) Cell migration was quantified by cell counting. Five random high‐power fields (HPFs) were selected in each well (**p < 0.01). g) In vivo evaluation of neovascularization. Blood perfusion in each group was detected by LDI at different time points (*n* = 3). Implantation sites are circled. Relative flux intensity was calculated using LDI system software (**p < 0.01). Mice were sacrificed on day 14, and samples were prepared for immunofluorescence staining of CD31 and α‐SMA. Six random HPFs were selected in each group for the calculation of blood vessel area (*n* = 6, **p < 0.01).

The process of bone formation is coupled to the process of angiogenesis.^9^ Blood vessels invade into the ossification region to initiate osteogenesis.^10^ Several chemokines, such as vascular endothelial growth factor (VEGF), stromal cell‐derived factor‐1 (SDF‐1), and platelet‐derived growth factor (PDGF), have been recognized to play important role in angiogenesis.^23^ In this study, we investigated whether the Mg‐enriched environment could upregulate the expression of those chemokines above in BMSCs. Results showed that after 2 days of incubation, enhanced expression of VEGF was detected in BMSCs encapsulated in Mg‐enriched spheroids, while the expressions of SDF‐1 and PDGF remained unchanged. It indicates that Mg‐enriched environment might promote angiogenesis via upregulating the expression of VEGF in BMSCs. Based on this finding, a transwell migration model was used to detect the in vitro recruitment capacity of the Mg‐enriched culture system in ECs. As shown in **Figure** **5**d, different groups of spheroids were cultured in the lower chambers for 2 days: SA microspheres without cells (SA), SA microspheres encapsulating BMSCs (SA‐BMSCs), and cell‐laden SA microspheres with a local Mg^2+^ concentration of 5 × 10^−3^ m (SA‐Mg/BMSCs). Then, the ECs were suspended in medium and seeded in the upper chambers, and EC migration was observed at different time points within 24 h. It was clear that in the groups without Mg^2+^, only limited cell responses occurred after incubation for 12 h, and no significant increase in migration was observed at 24 h. In the Mg‐enriched group, obvious cell responses occurred at early time points of incubation, and the maximum response was observed after incubation for 24 h (Figure 5e). The cell counts confirmed the significant differences in the migration of ECs between the Mg‐enriched group and the other two groups (Figure 5f). We further evaluated the angiogenic effects in vivo. After subcutaneous implantation of spheroids in nude mice, blood perfusion was detected over time by laser Doppler imaging (LDI) (Figure 5g). The relative flux intensity was calculated using the LDI image analysis software. At day 3, the early time point, the blood perfusion in the SA‐BMSC group was slightly higher than that in the SA group but lower than that in the SA‐Mg/BMSC group. At this time point, the difference between the SA‐Mg/BMSC group and the SA group was already significant. Although blood perfusion increased at later time points in both the SA‐BMSC and SA‐Mg/BMSC groups, the highest flux intensity was found in the SA‐Mg/BMSC group, and there was a significant difference between the SA‐Mg/BMSC group and the other groups from day 7 onward. At day 14, the degree of blood perfusion in the SA‐Mg/BMSC group increased to nearly three times that in the SA group. Immunohistochemistry analysis of frozen sections showed the presence of more CD31‐positive and α‐smooth muscle actin (α‐SMA)‐positive vessels in the SA‐Mg/BMSC group, and the blood vessel area was significantly different between the SA‐Mg/BMSC group and the other two groups. In short, a Mg‐enriched environment stimulated neovascularization from days 3 to 14. Previously we found that Mg^2+^ released from scaffolds could directly regulate the migration of ECs to defect region then facilitate neovascularization.^6^ Based on this finding, we here hypothesize that Mg^2+^ from the Mg‐enriched spheroids might be involved in the process of angiogenesis as well. In conclusion, Mg‐enriched environment may enhance angiogenesis through directly stimulating the migration of ECs and via upregulating the expression of VEGF in BMSCs. Furthermore, the fibronectin‐derived adhesion peptide arginine glycine aspartic acid (RGD) and its subtypes are commonly used to improve the cell adhesion to alginate. Studies indicate that RGD‐conjugated alginate not only improves the proliferation of ECs, but also stimulates osteogenic differentiation of stem cells. Introducing the RGD peptide to Mg‐enriched SA spheroids may further improve the vascularized bone regeneration.^24^

### 2.7 In Vivo Evaluation of Vascularized Bone Regeneration Promoted by Mg‐Enriched Spheroids

**Figure 6:**
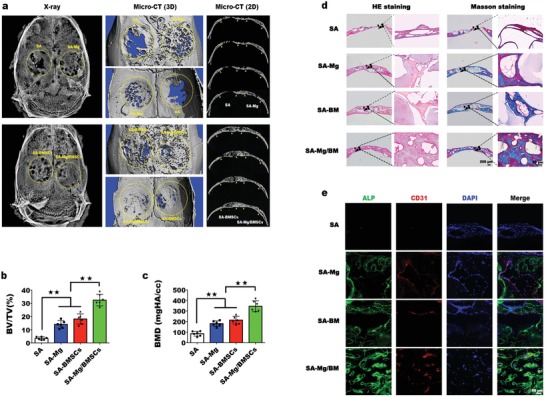
In vivo evaluation of vascularized bone regeneration using the rat cranial defects model. a) The graph shows the radiological analysis of samples collected 4 weeks after implantation. The yellow area indicates the defect area (*n* = 6). b,c) Statistical analysis of newly formed bone in the study groups (**p < 0.01). d) Decalcified sections were stained with H&E and Masson trichrome, and newly formed bone tissues appear blue. e) Costaining of CD31 and ALP revealed significant vascularized bone regeneration in the SA‐Mg/BM group.

In this section, the effects of Mg‐enriched spheroids on vascularized bone regeneration were investigated using the rat cranial defects model. The samples underwent radiological and histological analyses 4 weeks after implantation. **Figure** **6**a shows the results of the radiological analysis of bone formation. The SA‐Mg/BMSC group exhibited the best bone regeneration in all groups, with a large number of new bones filling in the defects, while in the SA group, only a small amount of new bone was observed. The regenerative effect in the SA‐Mg group was identical to that in the SA‐BMSC group. The 2D sectional micro‐computed tomography (micro‐CT) images showed that the normal structure of the skull was completely reconstructed in only the SA‐Mg/BMSC group. In the SA group, fibrous tissues without new bone formation was observed in most of the defect region, as evidenced by hematoxylin and eosin (H&E) staining (Figure 6d). Moreover, both in the SA‐BMSC and SA‐Mg groups, the newly formed bone structure was disrupted by undegraded spheroids, while there were fewer undegraded spheroids in the SA‐Mg/BMSCs group. Previous studies indicated that the bioactivities of cells encapsulated in gel and the phosphate ions could accelerate the degradation of alginate.^25^ Therefore, we deduced that the enhanced proliferation and ALP activity (leading to increases in phosphate ions) of BMSC encapsulated in Mg‐enriched spheroids contributed to a faster degradation of SA microspheres. Masson trichrome‐stained sections (Figure 6d) revealed that the cortical bone in the SA‐Mg/BMSC group was almost completely regenerated, as evidenced by the large consecutive blue areas, but this did not occur in the other groups. Furthermore, neovascularization in the different groups was evaluated by immunofluorescence staining of ALP and CD31, and the results are shown in Figure 6e. The total ALP‐positive area and number of CD31‐positive vessels were quite different in the SA‐Mg/BMSC group than in the other groups, implying the promotion of vascularized bone regeneration in the SA‐Mg/BMSC group. Furthermore, according to the quantitative micro‐CT analysis (Figure 6b,c), the bone volume/tissue volume (BV/TV) value of the SA‐Mg/BMSC group (32.425% ± 4.337%) was significantly higher than that of the SA‐BMSC group (18.148% ± 3.911%) and the SA‐Mg group (14.005% ± 2.864%), and this value was considerably lower in the SA group (3.492% ± 1.103%). Similar results were observed by comparing the bone mineral density (BMD) values, which indicate mineralization of new bone, in the different groups. Although the incorporation of Mg^2+^ or BMSCs separated into scaffolds promoted bone regeneration to some extent, the Mg‐enriched spheroids achieved a better regenerative effect by mimicking the bone development microenvironment.

## 3 Conclusion

In summary, inspired by the discovery that the expression of MagT1, a major Mg transporter, is significantly upregulated during bone development in mouse embryos, we successfully constructed a Mg‐enriched 3D culture system for stem cell delivery and bone regeneration. Upon the influx of Mg^2+^ via MagT1 and the subsequent activation of the MAPK/ERK pathway, the Mg‐enriched microenvironment remarkably enhanced the proliferation, migration, and osteogenic differentiation of encapsulated BMSCs and had clear effects on angiogenesis. More importantly, when the Mg‐enriched cell delivery complexes were implanted in rat critical‐sized skull defects, significant vascularized bone regeneration was achieved after 4 weeks. Our results indicate that the construction of biomaterials that mimic the developmental microenvironment might provide a tool to achieve better regenerative effects.

## 4 Experimental Section

*Animals*: Animals used in this study were provided by the Ninth People's Hospital Animal Center (Shanghai, China). All experiments and related protocols performed in this study were approved by the Animal Care and Experiment Committee of the Ninth People's Hospital.

*BMSC Isolation and Culture*: Rat BMSCs were obtained from the femurs and tibias of 3 week old Sprague Dawley rats as previously described.^26^ Cells were cultured in high‐glucose Dulbecco's modified Eagle medium (DMEM) (HyClone, USA) supplemented with 10% fetal bovine serum (FBS, Gibco, USA), 100 U mL^−1^ streptomycin, and 100 U mL^−1^ penicillin at 37 °C in an atmosphere of 5% CO_2_. Cells at passages 2–4 and 80–90% confluency were used for the in vitro and in vivo experiments.

*In Vivo and In Vitro Evaluation of MagT1 Expression during Osteogenic Differentiation*: Mouse embryos were collected at E14.5 and fixed for 24 h. The samples were then embedded in paraffin and sliced for histological evaluation. Immunofluorescence staining of multiple proteins was performed according to the manufacturer's instructions. Briefly, specific primary antibodies against ALP (R&D Systems, USA) and MagT1 (Proteintech Group, Inc., USA) were applied before using donkey anti‐goat Immunoglobulin G (IgG) (Yeasen, China) and donkey anti‐rabbit IgG secondary antibodies (Yeasen, China). The CLSM (Leica, Germany) was used for subsequent observation, and the remaining sections were stained with H&E.

In vitro osteogenic induction was carried out using osteogenic medium (Cyagen Biosciences, Inc., USA). BMSCs were cultured in osteogenic medium or normal medium (control). Total RNA was extracted with RNAiso Plus (TaKaRa, Japan), and complementary DNA (cDNA) was synthesized using the PrimeScript 1st Strand cDNA Synthesis kit (TaKaRa, Japan). The expression of MagT1 was detected using reverse transcription PCR (RT‐PCR), and amplification products were examined by 1.5% agarose gel electrophoresis. The results were confirmed by real‐time qPCR (Roche, Switzerland). The housekeeping gene glyceraldehyde‐3‐phosphate dehydrogenase (GAPDH) was used for normalization. Primer sequences are displayed in Table S1 (Supporting Information). Primary antibodies targeting OCN (Abcam, UK) and MagT1 (Proteintech Group, Inc., USA) were used for co‐immunofluorescence.

*Optimizing the Mg^2+^ Concentration for Osteogenic Induction*: To evaluate the effect of Mg^2+^ on cell viability, the IC_50_ of Mg^2+^ was determined. Briefly, DMEM with 1.6 × 10^−3^, 2.5 × 10^−3^, 5 × 10^−3^, 10 × 10^−3^, 20 × 10^−3^, 40 × 10^−3^, 60 × 10^−3^, or 80 × 10^−3^ m Mg^2+^ was created using MgCl_2_ (Sigma, USA). Normal DMEM with 0.8 × 10^−3^ m Mg^2+^ was used as a control. BMSCs were seeded in 96‐well plates at a density of 5000 cells per well. When the cells reached 90–95% confluence, the culture medium was replaced with the aforementioned high Mg medium (*n* = 6). After incubation for 24 h, 3‐(4,5‐dimethyl‐2‐thiazolyl)‐2,5‐diphenyl tetrazolium bromide (MTT) assays were conducted as previously described.^27^ The cell survival rate was calculated by comparing the optical density (OD) at 490 nm (OD_490_) of the Mg‐enriched groups with the control group. Cell viability was further confirmed by flow cytometry using an Annexin V‐FITC Apoptosis Detection Kit (BD Biosciences, USA) following the manufacturer's instructions. To optimize the Mg concentration for osteogenic differentiation, BMSCs were cultured in medium containing a series of Mg^2+^ concentrations. First, MagT1 expression after 5 days in culture was investigated by qPCR and immunofluorescence staining (*n* = 3). Actin and nuclei were stained with fluorescein isothiocyante (FITC)‐phalloidin (Yeasen, China) and 4′,6‐diamidino‐2‐phenylindole (DAPI) (Sigma, USA), respectively. After incubation for 5 days, BMSCs were fixed and stained with an ALP Staining kit (Beyotime, China), and semiquantitative analysis was conducted according to the manufacturer's instructions. Briefly, the sample was mixed with p‐nitrophenyl phosphate (p‐NPP) working solution (Beyotime, China) at 37 °C, and the OD_405_ was measured. Total protein was extracted from each sample and measured by the bicinchoninic acid (BCA) method (Thermo Fisher Scientific, USA). ALP activity was determined in a semiquantitative manner and reported based on the OD_405_ value per milligram total protein. Moreover, OCN protein levels were detected by western blot and immunofluorescence staining after 5 days of incubation. Total protein was extracted, separated by sodium dodecyl sulfate–polyacrylamide gel electrophoresis (SDS‐PAGE) and transferred to polyvinylidene fluoride membranes, which were incubated with primary antibody against OCN (Santa Cruz Biotechnology, USA) overnight at 4 °C. Secondary antibody (diluted 1:5000) was applied for 1 h at room temperature, and the membranes were treated with a chemoluminescence reagent (Thermo Fisher Scientific, USA) prior to exposure. The gray level of GAPDH was used for normalization. Immunofluorescence staining was carried out as described above.

*Analysis of the Possible Molecular Basis of Osteogenic Induction by Mg*: BMSCs were cultured in DMEM with 5 × 10^−3^ m Mg^2+^. Samples were collected at 0, 30, and 60 min to detect the phosphorylation of MAPK pathway components. Primary antibodies targeting p‐Erk1/2 (CST, USA), Erk1/2 (CST, USA), p‐P38 (CST, USA), P38 (CST, USA), p‐JNK (CST, USA), and JNK (CST, USA) were used in this study. Furthermore, BMSCs were cultured in medium containing 5 × 10^−3^ m Mg^2+^ and 10 × 10^−6^ m PD98059 (CST, USA). BMSCs cultured in high Mg medium without PD98059 were used as the control group. Erk1/2 phosphorylation was detected by western blot, and osteogenic differentiation was evaluated based on OCN expression and ALP activity.

*Targeted Disruption of the MagT1 Gene*: The CRISPR/Cas9 system was designed by Genomeditech (China). Lentiviral transfection was conducted according to the manufacturer's instructions. Briefly, BMSCs were seeded in 6‐well culture plates and then infected with lentivirus harboring antibiotic resistance at a multiplicity of infection (MOI) of 20 in the presence of 5 µg mL^−1^ polybrene. Blasticidin (10 µg mL^−1^) and puromycin (5 µg mL^−1^) were used to select positive cells. Mutation of the targeted gene was identified by gene sequencing (Genomeditech, China).

*Measurement of Intracellular Mg^2+^ in BMSCs*: Mg^2+^ entry into BMSCs was measured as previously described.^28^ Briefly, cells were incubated in medium supplemented with 2 × 10^−6^ m Mg‐Fura‐2 (Thermo Fisher Scientific, USA) for 30 min before being washed with physiological saline (0.9% NaCl). After incubation for another 30 min, the cells were moved to the CLSM platform (Leica, Germany) for observation of changes in fluorescence signals, and pictures were taken at 1 s intervals.

*Osteogenic Differentiation Analysis of MagT1‐Knockout BMSCs in a Mg‐Enriched Environment*: After MagT1 knockout, BMSCs were cultured in medium with 5 × 10^−3^ m Mg^2+^. Normal BMSCs (BMSCs that were transfected by lentivirus carrying negative control) under the same conditions were used as controls. Activation of the MAPK/ERK pathway was investigated by western blot, and osteogenic differentiation was measured based on ALP activity.

*Fabrication and Optimization of the Mg‐Enriched 3D Culture System*: SA powder (Sigma, USA, product number is 180 947) was dissolved in physiological saline at a concentration of 4% w/v and stirred overnight. BMSCs were suspended in medium with a high Mg^2+^ concentration. After autoclaving, the alginate solution was mixed with a BMSC suspension, and the final Mg^2+^ concentration of the mixture was adjusted to 2.5 × 10^−3^, 5 × 10^−3^, or 0.8 × 10^−3^ m as a control. The spheroids were fabricated by dropping the mixture into a 0.1 m CaCl_2_ solution. After crosslinking for 2 min, the cell‐encapsulated spheroids were washed three times with physiological saline and then cultured in DMEM for subsequent experiments. Spheroids with a diameter of 500 µm were created using a microliter syringe (Hamilton, Switzerland), while larger spheroids were made with a normal syringe (BD Biosciences, USA). Live/Dead assays were conducted using the Calcein‐AM/PI Double Stain Kit (Yeasen, China). Live cells stained by Calcein‐AM emit green fluorescence, while PI‐positive dead cells have red fluorescence. After being cultured for 1 and 3 days, spheroids were dissolved in 55 × 10^−3^ m sodium citrate solution. The viability of cells released from spheroids was detected by a CCK‐8 kit (Dojindo, Japan) according to the manufacturer's instructions, as previously reported.^29^

*In Vitro Osteogenic Differentiation Analysis of Encapsulated BMSCS*: To evaluate the proliferation of encapsulated BMSCs, spheroids were cultured in medium supplemented with EdU (diluted 1:1000, 1 mg L^−1^; Ribobio, China). About 50 µL of spheroids from each group was collected and mounted in OCT embedding compound for frozen sections. Proliferating cells were labeled by EdU. Sections were stained following the manufacturer's instructions. Briefly, sections were incubated with EdU staining working solution for 30 min at room temperature in the dark. Cell nuclei were counterstained with DAPI (Sigma, USA) for 5 min. Slides were observed by fluorescence microscopy (Olympus, Japan). The percentage of EdU‐positive cells was calculated. To investigate the migration of encapsulated cells, BMSCs were labeled with GFP before being encapsulated in spheroids. 3D reconstruction images were obtained by a CLSM (Leica, Germany). The expression levels of ALP, Runx2, Osx, and OCN in BMSCs released from spheroids were examined by qPCR at days 3 and 5 of culture.

*In Vivo and In Vitro Detection of the Angiogenic Effects of the Mg‐Enriched Microenvironment*: Each group in this experiment was analyzed in at least three replicates. For the transwell migration model, spheroids were cultured in the lower chambers with DMEM for 2 days. Then, ECs were suspended in DMEM and seeded into the upper chambers. After 12 or 24 h, the upper chambers were removed, fixed in 4% paraformaldehyde, and stained with crystal violet. Five random high‐power fields (HPFs) were selected in each well to count cell numbers. Spheroids were implanted subcutaneously in nude mice. Blood perfusion was measured by LDI (Moor Instruments, UK) at 1, 3, 7, and 14 days after implantation. Relative flux intensity was calculated using LDI image analysis software. After 14 days of implantation, mice were sacrificed for histological examination. Samples were embedded in OCT to generate frozen sections, and subsequent immunofluorescence staining was performed to detect neovascularization. Primary antibodies against CD31 (R&D Systems, USA) and α‐SMA (Abcam, UK) were applied. Six random HPFs were selected in each group to calculate the blood vessel area. The expression of chemotactic factors, including PDGF, SDF‐1, and VEGF, in encapsulated BMSCS was measured using qPCR.

*In Vivo Bioactivity Analysis of the Mg‐Enriched 3D Culture System*: A rat critical‐sized cranial defects model was created as previously reported to evaluate vascularized bone regeneration.^26^ Two full thickness defects with a 5 mm diameter were made on both sides of the rat skull, and spheroids from the following four groups were implanted: SA spheroids (SA), SA spheroids with 5 × 10^−3^ m Mg^2+^ (SA‐Mg), cell‐laden spheroids (SA‐BMSCs), and cell‐laden spheroids with 5 × 10^−3^ m Mg^2+^ (SA‐Mg/BMSCs). Six samples of each group were analyzed in the experiment. Rats were sacrificed 4 weeks after surgery, and tissues were fixed for 24 h. Then, the samples were scanned by an X‐ray imaging system (Faxitron, USA) and a micro‐CT system (µCT50, Scanco Medical, Switzerland), and 3D reconstruction and quantitative analysis of bone regeneration were carried out using the micro‐CT system software. After immersion in 20% ethylenediaminetetraacetic acid (EDTA) solution for 20 days, decalcified samples were prepared for subsequent histological examination. H&E and Masson trichrome staining (Leagene, China) were performed according to the manufacturer's instructions. To further evaluate neovascularization, immunofluorescence costaining of ALP (R&D Systems, USA) and CD31 (Novus Biologicals, USA) was conducted following the aforementioned steps.

*Statistical Analysis*: All data were presented as the mean ± standard deviation. The statistical analysis was performed using the SAS 8.2 statistical software package. Comparisons between two groups were analyzed by Student's *t*‐test (*p < 0.05, **p < 0.01); otherwise, one‐way analysis of variance (ANOVA) was performed, followed by Tukey's posthoc test for multiple comparisons.

## Conflict of Interest

The authors declare no conflict of interest.

## Supporting information

Supplementary

Supplementary

Supplementary

## References

[1] a) M. Stevens , Mater. Today 2008, 11, 18;

[2] A. Simon , E. Tanaka , WIREs Dev. Biol. 2013, 2, 291. 10.1002/wdev.73 24009038

[3] a) T. Gerber , P. Murawala , D. Knapp , W. Masselink , M. Schuez , S. Hermann , M. Gac‐Santel , S. Nowoshilow , J. Kageyama , S. Khattak , J. Currie , J. Camp , E. Tanaka , B. Treutlein , Science 2018, 362, eaaq0681; 30262634 10.1126/science.aaq0681 PMC6669047

[4] S. Bose , M. Roy , A. Bandyopadhyay , Trends Biotechnol. 2012, 30, 546. 22939815 10.1016/j.tibtech.2012.07.005 PMC3448860

[5] a) H. Cui , W. Zhu , B. Holmes , L. Zhang , Adv. Sci. 2016, 3, 1600058; 10.1002/advs.201600058 PMC5074245 27818910

[6] W. Zhang , C. Feng , G. Yang , G. Li , X. Ding , S. Wang , Y. Dou , Z. Zhang , J. Chang , C. Wu , X. Jiang , Biomaterials 2017, 135, 85. 28499127 10.1016/j.biomaterials.2017.05.005

[7] L. Roseti , V. Parisi , M. Petretta , C. Cavallo , G. Desando , I. Bartolotti , B. Grigolo , Mater. Sci. Eng., C 2017, 78, 1246. 10.1016/j.msec.2017.05.017 28575964

[8] S. Bose , G. Fielding , S. Tarafder , A. Bandyopadhyay , Trends Biotechnol. 2013, 31, 594. 24012308 10.1016/j.tibtech.2013.06.005 PMC3825404

[9] K. Sivaraj , R. Adams , Development 2016, 143, 2706. 27486231 10.1242/dev.136861

[10] S. Stegen , N. van Gastel , G. Carmeliet , Bone 2015, 70, 19. 25263520 10.1016/j.bone.2014.09.017

[11] Y. Yu , G. Jin , Y. Xue , D. Wang , X. Liu , J. Sun , Acta Biomater. 2017, 49, 590. 27915020 10.1016/j.actbio.2016.11.067

[12] F. Li , B. Chaigne‐Delalande , C. Kanellopoulou , J. Davis , H. Matthews , D. Douek , J. Cohen , G. Uzel , H. Su , M. Lenardo , Nature 2011, 475, 471. 21796205 10.1038/nature10246 PMC3159560

[13] H. Zhou , D. Clapham , Proc. Natl. Acad. Sci. USA 2009, 106, 15750. 19717468 10.1073/pnas.0908332106 PMC2732712

[14] a) G. Wang , J. Li , W. Zhang , L. Xu , H. Pan , J. Wen , Q. Wu , W. She , T. Jiao , X. Liu , X. Jiang , Nanomedicine 2014, 9, 2387; 24940056 10.2147/IJN.S58357 PMC4051717

[15] a) J. Wang , X. Ma , Y. Feng , Z. Ma , T. Ma , Y. Zhang , X. Li , L. Wang , W. Lei , Biol. Trace Elem. Res. 2017, 179, 284; 28205079 10.1007/s12011-017-0948-8

[16] A. James , Scientifica 2013, 2013, 1.

[17] a) F. Topuz , A. Henke , W. Richtering , J. Groll , Soft Matter 2012, 8, 4877;

[18] M. Laschke , M. Menger , Trends Biotechnol. 2017, 35, 133. 27634310 10.1016/j.tibtech.2016.08.004

[19] G. Whitesides , B. Grzybowski , Science 2002, 295, 2418. 11923529 10.1126/science.1070821

[20] K. Groebe , W. Mueller‐Klieser , Int. J. Radiat. Oncol., Biol., Phys. 1996, 34, 395. 8567341 10.1016/0360-3016(95)02065-9

[21] a) Y. Man , P. Wang , Y. Guo , L. Xiang , Y. Yang , Y. Qu , P. Gong , L. Deng , Biomaterials 2012, 33, 8802; 22981779 10.1016/j.biomaterials.2012.08.054

[22] X. Zhao , S. Liu , L. Yildirimer , H. Zhao , R. Ding , H. Wang , W. Cui , D. Weitz , Adv. Funct. Mater. 2016, 26, 2809.

[23] a) N. Ferrara , H. Gerber , J. LeCouter , Nat. Med. 2003, 9, 669; 12778165 10.1038/nm0603-669

[24] a) C. Chun , H. Lim , K. Hong , K. Park , S. Song , Biomaterials 2009, 30, 6295; 19712969 10.1016/j.biomaterials.2009.08.011

[25] a) S. Takka , F. Acarturk , J. Microencapsulation 1999, 16, 291; 10340215 10.1080/026520499289022

[26] W. Zhang , Q. Chang , L. Xu , G. Li , G. Yang , X. Ding , X. Wang , D. Cui , X. Jiang , Adv. Healthcare Mater. 2016, 5, 1299. 10.1002/adhm.201500824 26945787

[27] W. Zhang , L. Wray , J. Rnjak‐Kovacina , L. Xu , D. Zou , S. Wang , M. Zhang , J. Dong , G. Li , D. Kaplan , X. Jiang , Biomaterials 2015, 56, 68. 25934280 10.1016/j.biomaterials.2015.03.053

[28] Y. Zhang , J. Xu , Y. Ruan , M. Yu , M. O'Laughlin , H. Wise , D. Chen , L. Tian , D. Shi , J. Wang , S. Chen , J. Feng , D. Chow , X. Xie , L. Zheng , L. Huang , S. Huang , K. Leung , N. Lu , L. Zhao , H. Li , D. Zhao , X. Guo , K. Chan , F. Witte , H. Chan , Y. Zheng , L. Qin , Nat. Med. 2016, 22, 1160. 27571347 10.1038/nm.4162 PMC5293535

[29] C. Feng , W. Zhang , C. Deng , G. Li , J. Chang , Z. Zhang , X. Jiang , C. Wu , Adv. Sci. 2017, 4, 1700401. 10.1002/advs.201700401 PMC5737106 29270348

