# Supplementary material for: A Magnesium‐Enriched 3D Culture System that Mimics the Bone Development Microenvironment for Vascularized Bone Regeneration
Source: Adv Sci (Weinh). 2019 Apr 18;6(12):1900209. doi: 10.1002/advs.201900209 (PMC6662069; doi:10.1002/advs.201900209)
Supplement: Supplementary file 1 — Supplementary [file ADVS-6-1900209-s002.pdf]

Copyright WILEY-VCH Verlag GmbH & Co. KGaA, 69469 Weinheim, Germany, 2019.

ADVANCED SCIENCE
Open Access

Supporting Information

for *Adv. Sci.,* DOI: 10.1002/advs.201900209

A Magnesium-Enriched 3D Culture System that Mimics the Bone Development Microenvironment for Vascularized Bone Regeneration

Sihan Lin, Guangzheng Yang, Fei Jiang, Mingliang Zhou, Shi Yin, Yanmei Tang, Tingting Tang, Zhiyuan Zhang, Wenjie Zhang, and Xinquan Jiang**

Copyright WILEY-VCH Verlag GmbH & Co. KGaA, 69469 Weinheim, Germany, 2019.

Supporting Information

A magnesium-enriched 3D culture system that mimics the bone development microenvironment for vascularized bone regeneration

Sihan Lin[#], Guangzheng Yang[#], Fei Jiang, Mingliang Zhou, Shi Yin, Yanmei Tang, Tingting Tang, Zhiyuan Zhang, Wenjie Zhang[*], Xinquan Jiang[*]

S. Lin, G. Yang, F. Jiang, M. Zhou, S. Yin, Y. Tang, Dr. W. Zhang, Prof. X. Jiang
Department of Prosthodontics
Shanghai Engineering Research Center of Advanced Dental Technology and Materials
Shanghai Research Institute of Stomatology
National Clinical Research Center for Oral Diseases
Shanghai Key Laboratory of Stomatology
Ninth People's Hospital, College of Stomatology, Shanghai JiaoTong University School of Medicine
639 Zhizaoju Road, Shanghai 200011, China
E-mail: xinquanj@aliyun.com; zhangwenjie586@126.com

Prof. T. Tang
Department of Orthopaedic Surgery
Ninth People's Hospital affiliated to Shanghai JiaoTong University School of Medicine
639 Zhizaoju Road, Shanghai 200011, P. R. China

Prof. Z. Zhang.
Department of Oral and Maxillofacial-Head and Neck Oncology
Shanghai Research Institute of Stomatology
National Clinical Research Center for Oral Diseases
Shanghai Key Laboratory of Stomatology
Ninth People's Hospital, College of Stomatology, Shanghai JiaoTong University School of Medicine
639 Zhizaoju Road, Shanghai 200011, P. R. China

[#] These authors contributed equally.

1. Experimental section

Analysis of BMSC proliferation in medium with a gradient concentration of Mg^{2+}

Gradient concentrations of Mg^{2+} (1.6 mM, 2.5 mM, 5 mM, 10 mM, and 20 mM) were created by adding $MgCl_2$ (Sigma, USA) to high-glucose DMEM (HyClone, USA). Normal DMEM was used as a control. Briefly, BMSCs were seeded in 96-well plates at a density of 1000 cells per well. Medium with different concentrations of Mg^{2+} was added 12 hours later. Six replicates in each group were analyzed in this experiment. The MTT assay was conducted after 1, 4, and 7 days of incubation.

2. Supporting figures

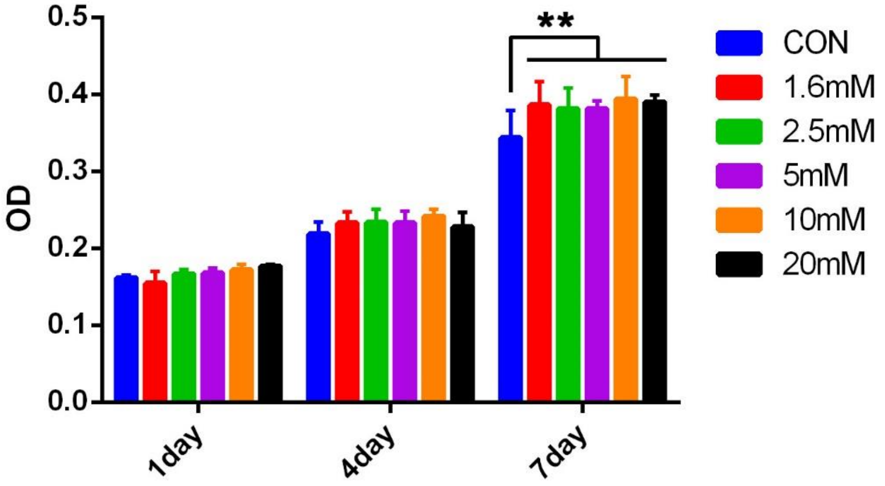

Figure S1. MTT assay of cell proliferation in different magnesium environment.

3. Supporting table

Table S1. Primer sequences for qPCR.

Gene	Forward primer	Reverse primer
GAPDH	GTCTCCTCTGACTTCAACAGCG	ACCACCCTGTTGCTGTAGCC
MagT1	TGACATGTTTTCAAGCTTAGGC	GACCAGCTTTCAAGGAACAATT
ALP	CCCACAAGAGCCCACAAT	AGAGCCAGGAATCCGACCC
Osx	TCCTGCGACTGCCCTAATTGC	TCCGAACGAGTGAACCTCTTGC
Runx2	ACCAGCAGCACTCCATATCTCTAC	CTTCCATCAGCGTCAACACCATC
OCN	ATTGTGACGAGCTAGCGGAC	GCAACACATGCCCTAAACGG
PDGF	TCTCTGCTGCTACCTGCGTCTG	AAGGAGCGGATGGAGTGGTCAC
SDF-1	ACCTCGGTGTCCTCTTGCTG	GATGTTTGACGTTGGCTCTGG
VEGF	GGCTCTGAAACCATGAACTTTCT	GCAGTAGCTGCGCTGGTAGAC

4. Supplementary files

File S1. Result of Gene sequencing of MagT1 in BMSCs infected by lentivirus carrying negative control.

File S2. Result of Gene sequencing of MagT1 in BMSCs infected by lentivirus carrying sgRNA.

5. Supplementary movies

Movie S1. A remarkable increase of intracellular fluorescence intensity resulting from addition of Mg^{2+}.

Movie S2. The slightly change in intracellular fluorescence intensity indicates the inhibitory effects of knockout of MagT1 on uptake of Mg^{2+} in BMSCs.
